# Blast sampling for structural and functional analyses

**DOI:** 10.1186/1471-2105-8-62

**Published:** 2007-02-23

**Authors:** Anne Friedrich, Raymond Ripp, Nicolas Garnier, Emmanuel Bettler, Gilbert Deléage, Olivier Poch, Luc Moulinier

**Affiliations:** 1 Laboratoire de Bioinformatique et Génomique Intégratives, Institut de Génétique et de Biologie Moléculaire et Cellulaire, Illkirch, France; 2 Institut de Biologie et Chimie des Protéines, Lyon, France

## Abstract

**Background:**

The post-genomic era is characterised by a torrent of biological information flooding the public databases. As a direct consequence, similarity searches starting with a single query sequence frequently lead to the identification of hundreds, or even thousands of potential homologues. The huge volume of data renders the subsequent structural, functional and evolutionary analyses very difficult. It is therefore essential to develop new strategies for efficient sampling of this large sequence space, in order to reduce the number of sequences to be processed. At the same time, it is important to retain the most pertinent sequences for structural and functional studies.

**Results:**

An exhaustive analysis on a large scale test set (284 protein families) was performed to compare the efficiency of four different sampling methods aimed at selecting the most pertinent sequences. These four methods sample the proteins detected by BlastP searches and can be divided into two categories: two customisable methods where the user defines either the maximal number or the percentage of sequences to be selected; two automatic methods in which the number of sequences selected is determined by the program. We focused our analysis on the potential information content of the sampled sets of sequences using multiple alignment of complete sequences as the main validation tool. The study considered two criteria: the total number of sequences in BlastP and their associated E-values. The subsequent analyses investigated the influence of the sampling methods on the E-value distributions, the sequence coverage, the final multiple alignment quality and the active site characterisation at various residue conservation thresholds as a function of these criteria.

**Conclusion:**

The comparative analysis of the four sampling methods allows us to propose a suitable sampling strategy that significantly reduces the number of homologous sequences required for alignment, while at the same time maintaining the relevant information concerning the active site residues.

## Background

Recent developments in whole genome sequencing, assembly techniques and expressed sequence tag (EST[1]) methods have lead to a vast amount of sequence data flooding the protein and DNA databases. Over 390 complete genomes are now referenced on the GOLD[2] web site[3] with many others in the sequencing and assembly stages. In addition, the recent emergence of high throughput functional genomics techniques has increased the rate at which genome and sequence products are being functionally characterized. As a consequence, the majority of new sequences have homologues in the public databases, and new functional or structural data may often be inferred for the sequence under study using database mining.

Thus, sequence database mining and analysis have become essential first steps for a wide range of applications in molecular biology. One of the most widely used methods for detecting homologous sequences is Blast[4]. The Blast suite of programs is used to find local sequence similarities, which might lead to evolutionary clues about the structure and/or function of the query sequence. The detected sequences can then be used e.g. to build a multiple alignment of complete sequences (MACS), which represents an ideal workbench to study all the information related to a set of homologous sequences[5]. Indeed, by placing a sequence in the context of its overall family, the MACS permits not only a "horizontal" analysis of the sequence along its complete length, but also a "vertical" view of its evolution among different organisms. MACS are typically used to perform comparative analyses at the genome level, to define the phylogenetic relationships between organisms in evolutionary studies, to identify conserved functional residues, motifs or domains and to predict protein or RNA secondary and tertiary structures[6].

As a direct consequence of the recent database growth, Blast searches frequently lead to the identification of hundreds to thousands of potential homologues for a single query sequence. Dealing with so much data can be detrimental, not only in terms of computational and human analysis time, but also in terms of the accuracy and the significance of the results. Problems, such as sequencing or intron/exon prediction errors, redundancy or the presence of partial sequences, may represent a significant source of noise, depending on the biological question under study.

It is therefore essential to develop novel strategies to reduce the set of sequences to be processed at the earliest possible stage of an analysis, which is generally during the sequence database search. There are clearly two possibilities: an *a priori *reduction of the sequence database search space or an *a posteriori *sampling of the sequences detected by the database search program. Some types of studies have intrinsic *a priori *sequence filters; e.g. the construction of a phylogenetic distribution of proteins from complete genomes or the analysis of proteins belonging to specific clades. Another *a priori *strategy is the use of a pre-processed non-redundant database, where sequences are clustered by means of their percent identities, such as the UniRef database series[7] (UniRef90, UniRef50). The *a posteriori *sampling methods are generally based on sequence similarity criteria and frequently require user intervention. For example, UniqueProt[8] is a fast and simple method that reduces the redundancy of the dataset by removing over-represented sequences, based on a user-defined percent identity threshold. This method works reasonably well when the proteins have similar domain architectures. A similar strategy is incorporated in BLAST Filter[9], which generates smaller sequence sets by filtration of Blast results based on 15 distinct user-configurable rules requiring a complex pre-scanning of the Blast results. These methods are therefore not suitable for automatic, high-throughput projects. A more recent study describes a Monte-Carlo sequence selection strategy[10] to improve the detection of residues belonging to a functional surface in the context of a multiple alignment of proteins of known structure. However the latter study samples sequences after the construction of the multiple alignment, which may incur a large time penalty. If possible, it is clearly advantageous in terms of processing time, to address the relationship between sequence sampling and the information content of the resulting alignment during the initial Blast search step.

Here we analyse a number of different methods for the sampling of protein sequences detected by BlastP searches, aimed at significantly reducing the set of sequences to be processed, while maintaining the same information potential. We have considered sampling methods only based on E-values because this parameter integrates several factors such as sequence length, subject and query similarity scores, database size, although it would be theoretically possible to take into account other factors such as the matching sequences themselves or the species from which they are derived. Four methods have been studied (see *Methods*) which can be divided into two categories:

- two customisable methods with user-defined parameters that determine either the maximal number of sequences to be selected (the strips method, sm), or the percent reduction rate (the random method, rm),

- two automatic methods based only on the E-values calculated by BlastP: the mean method (mm) and the second derivative method (sdm). These methods automatically determine the number of sequences in the sampled set.

We focused our analysis on the potential information contained in the sampled sets of BlastP sequences, using the MACS as our main validation tool. Our analysis focused more precisely on the conservation of residues implicated in the active sites of 284 proteins with known and annotated 3D structure selected from the Protein Data Bank (PDB)[11]. A good sampling method should not alter the global quality of the resulting alignments, and should preserve the relevant structural and functional information, e.g. the conservation of active site residues. This analysis allows us to propose a suitable strategy to sample homologous sequences, while keeping the pertinent information in the associated MACS.

## Results and discussion

### Global strategy

**Figure 1:**
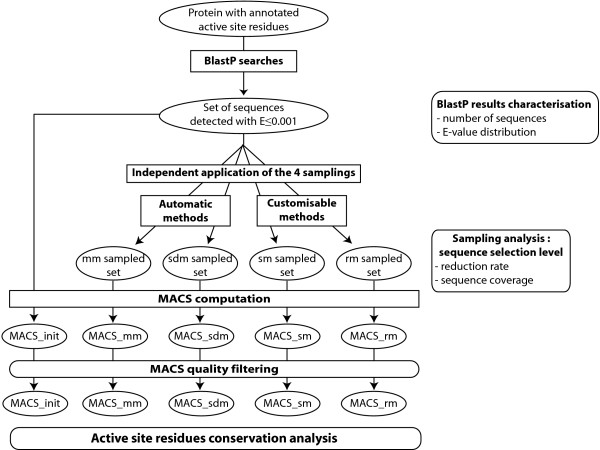
**Strategy flowchart**. For each protein in the initial 284 protein dataset, the set of potential homologous sequences was detected by BlastP searches. BlastP results were then characterised according to the number of sequences detected and their associated E-value distribution. The 4 sampling methods (2 automatic methods: the mean method mm and the second derivative method sdm; 2 customisable methods: the strips method sm and the random method rm) were independently applied to the initial set and analysed in terms of reduction rate properties and sequence coverage between the methods. Finally, the 5 associated multiple alignments of complete sequences (MACS) were computed. Taking into account the common high quality MACS, the variation of the information content of the sampled sets were studied, based on the conservation of the active site residues.

The multi-step process used to compare the different sampling methods is shown in Figure 1. For each protein in a large test set of 284 proteins:

- a BlastP search was performed in the Uniref90 database to identify the set of potential homologous sequences. Of the 284 BlastP searches, 36 detected more than 1000 sequences with E-value ≤ 0.001, which illustrates the necessity for new strategies that are capable of reducing the number of sequences to process in subsequent analyses.

- each sampling method (the mean method mm, the second derivative method sdm, the strips method sm and the random method rm) was independently applied to the set of detected sequences, resulting in a sampled sequence set containing a reduced number of sequences.

Five sets of sequences were thus associated with each initial protein: the unsampled set of sequences detected by BlastP and the 4 sampled sequence sets. These sets of sequences were then multiply aligned (when necessary, we limited the alignment to 500 sequences with the lowest E-values before or after sampling), resulting in five multiple alignments of complete sequences (MACS): respectively MACS_init containing the top 500 sequences detected by BlastP, MACS_mm, MACS_sdm, MACS_sm and MACS_rm.

The first part of the analysis studies the reduction rate associated with the different sampling methods, depending on the initial number of sequences in the BlastP results and their E-value distribution. We also studied the amount of sequence coverage between the different methods, in order to estimate their redundancy or complementarity. The second part then studies the effect of the sampling methods on the MACS information content by considering the quality of the MACS and the conservation of documented active site residues.

### Large scale comparison of sampled sequence sets

We studied the behaviour of the different sampling methods for a large set of diverse BlastP searches (concerning 284 protein families). We analysed the effect of the BlastP results on the ability of the sampling methods to effectively reduce the number of sequences according to two criteria: the total number of sequences detected and their E-value distribution. We also compared the sequence coverage between the different sampling methods.

#### Sequence reduction rate

For each protein in the 284 protein dataset, the evaluation of the reduction rate associated with each sampling method was based on the calculation of the ratio between the number of sequences obtained after sampling and the number of sequences in the initial BlastP result file with an E-value ≤ 0.001. For each sampling method, the mean reduction rate is then defined as the average of the 284 computed reduction rates. Surprisingly, the mean reduction rate obtained with the automatic methods mm and sdm is very similar to that obtained using the customisable method sm, with all methods resulting in approximately 70% reduction in the number of sequences (Table 1). These preliminary observations prompted us to set the reduction ratio associated with the customisable random method rm to 70%.

**Table 1:** Mean reduction rate associated with sampling methods

		**mm**	**sdm**	**sm**
**global (284 seq.)**	Mean reduction ratio (%)	70	70	71
	Standard deviation	14	14	26
**subset-100 (91 seq.)**	Mean reduction ratio (%)	60	60	42
	Standard deviation	17	17	23
**subset100–500 (114 seq.)**	Mean reduction ratio (%)	70	69	78
	Standard deviation	9	6	9
**subset+500 (79 seq.)**	Mean reduction ratio (%)	80	81	94
	Standard deviation	10	9	4

However, the standard deviation obtained with the sm (26) is approximately twice as large as that associated with the automatic methods (both 14), suggesting that the methods may have distinct behaviours depending on the BlastP results. Also, the total number of sequences selected by each method varies significantly. Of the total 153128 sequences detected with E-value ≤ 0.001, sm selects 12108 sequences, sdm 26676 sequences, mm 28061 sequences and rm 45992 sequences. Therefore, in order to gain more insight into the influence of the BlastP results on the behaviour of the sampling methods, we divided the 284 protein dataset into three subsets of comparable size according to the total number of sequences detected by BlastP with E-value ≤ 0.001:

- subset-100: 91 proteins for which BlastP detected less than 100 sequences

- subset100–500: 114 proteins for which BlastP detected between 100 and 500 sequences

- subset+500: 79 proteins for which BlastP detected more than 500 sequences

**Figure 2:**
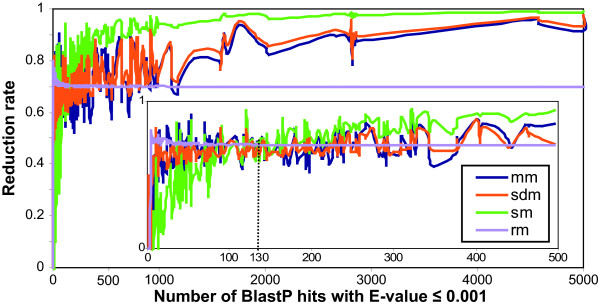
**Sampling methods reduction rate**. Sampling methods reduction rate plotted as a function of the number of sequences detected with an E-value ≤ 0.001 by BlastP searches. A zoom on the 0 to 500 detected sequence interval is shown, with the position 130 corresponding to the number of sequences detected by BlastP searches for which the reduction rate of sm become higher than the reduction rate of mm and sdm.

This division of the initial dataset shows that the number of sequences in the BlastP results file affects the mean reduction rate associated with all of the sampling methods tested (Table 1). A similar behaviour is observed for mm and sdm and both can be considered as progressive as their mean reduction rate increases almost linearly from 60 to 80% for subset-100 to subset+500. In contrast, the reduction rate obtained with sm is more variable: subset-100 is reduced by 42%, subset100–500 by 78% and subset+500 by 94%, with the standard deviations decreasing from subset-100 to subset+500. Figure 2 confirms that in general, the reduction rate increases with the number of sequences detected by BlastP for all the sampling methods (except rm, whose reduction rate was fixed at 70%). Moreover, the reduction obtained by mm and sdm is similar for all the tests in the 284 protein dataset. Closer investigation (see zoom in Figure 2) showed that, for BlastP results with less than about 130 sequences, the reduction rate of mm and sdm is higher than the sm reduction rate and for more than 130 sequences, the situation is reversed and sm obtains a higher reduction rate. There is clearly a relationship between the number of sequences detected by BlastP and the number of sequences in the sampled sets for all the methods (except rm). However, the reduction curves are not linear and we hypothesize that the number of sequences detected by BlastP searches is not the only parameter that influences the sampling reduction properties.

**Figure 3:**
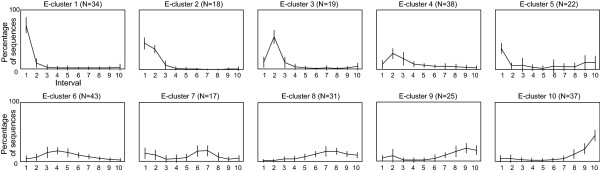
**BlastP E-value distribution**. Graphical representation of the 10 E-clusters representing the BlastP E-value distribution. E-cluster 1 corresponds to highly populated interval 1, i.e. a majority of highly related sequences in BlastP results. E-cluster 10 corresponds to highly populated interval 10, i.e. a majority of weakly related sequences in BlastP results. N represents the number of BlastP searches in each E-cluster.

**Figure 4:**
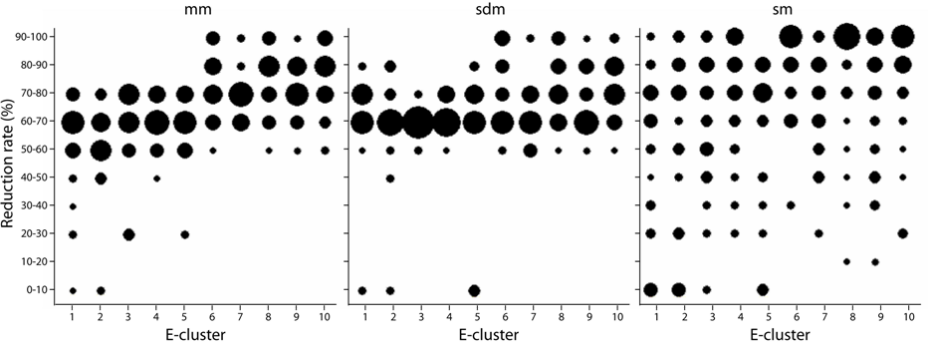
**Sampling methods reduction rate as a function of the E-clusters**. Sampling methods reduction rate plotted as a function of the distribution of the BlastP E-values (E-clusters). For a given E-cluster, the sizes of the black spots are proportional to the percentage of sampled BlastP at each reduction rate.

We therefore analysed the impact of the distribution of BlastP E-values on the sampling reduction rate. The 284 BlastP search results were categorized in 10 clusters (named E-clusters) according to the type of E-value distribution observed (see *Methods *and Figure 3 for more details). E-cluster 1 corresponds to BlastP results containing a majority of highly similar sequences and E-cluster 10 contains BlastP results with a majority of weakly related sequences. Figure 4 shows that the three sampling methods are not affected in the same way by the distribution of the BlastP E-values. The reduction rate obtained using mm clearly increases from E-cluster 1 to 10. This is due to the fact that, as mm is based on the difference between two successive log(E-value), the range of log(E-value) in each E-cluster will strongly influence the reduction rate. For example, E-cluster 1 is mainly composed of BlastP searches with E-values ranging from 1.10^-200 ^to 4.10^-67 ^while E-cluster 10 is mainly composed of sequences detected with E-values ranging from 2.10^-5 ^to 0.001. As a consequence, when BlastP searches detect a majority of weakly related sequences (E-value close to 0.001), mm selects very few sequences. For the sdm method, a surprising tendency is observed: regardless of the BlastP sequence distribution, reduction rates are mostly between 60 and 70% with a slight increase for E-clusters 8 to 10. Thus, we conclude that sdm is weakly influenced by the E-value distribution. This somewhat surprising result may be due to a biased composition of the databases or may reflect a particular characteristic of the BlastP E-value calculation. The sm reduction rate is highly variable for all the E-clusters and is in fact, more closely related to the number of sequences detected by BlastP. This behaviour can be explained by the pre-defined maximal number of sequences to be selected, which is equal to the number of strips (set to 100 in our study).

In summary, the reduction rate of mm depends on both the number of BlastP sequences and their E-value distribution. In contrast, sdm and sm depend mainly on the number of sequences. Nevertheless, sdm and sm behave differently in relation to the BlastP E-value distribution: the sdm reduction rate is relatively constant, while the sm reduction rate is much more variable.

#### Sequence coverage

As specified in the *Sequence reduction rate *section, 153128 sequences with an E-value ≤ 0.001 were detected by the 284 BlastP searches and the sampling methods resulted in different total numbers of sequences. As shown in Table 2, mm and sdm selected approximately the same number of sequences (28061 and 26676 respectively), whereas sm selected a smaller number of sequences (12108) and rm much more (45992). For the sequences specifically selected by only one sampling method, we observed a large difference between their proportions: 7% for sm, 12% for mm, 15% for sdm and an extreme value for the random method, 79%, reflecting a fundamentally different behaviour of this method.

**Table 2:** Sequence coverage between the sampling methods. The method in the first column is considered as the reference

			**2^nd ^method**				
**1^st ^method (reference)**	**specific**	**mm-sm-sdm-rm**	**mm**	**sdm**	**sm**	**rm**	**3^rd ^method**

**mm **(28061)	3383 (12%)	1938 (7%)		4783 (17%)			**sm**
				3230 (12%)	885 (3%)		**rm**
				**10076 (36%)**	2436 (9%)	1118 (4%)	**-**

**sdm **(26676)	4120 (15%)	1938 (7%)	4783 (18%)				**sm**
			3230 (12%)		261 (1%)		**rm**
			**10076 (38%)**		614 (2%)	1734 (7%)	**-**

**sm **(12108)	833 (7%)	1938 (16%)	4783 (40%)				**sdm**
			885 (7%)	261 (2%)			**rm**
			2436 (20%)	614 (5%)		362 (3%)	**-**

**rm **(45992)	**36464 (79%)**	1938 (4%)	885 (2%)				**sm**
			3230 (7%)		261 (1%)		**sdm**
			1118 (2%)	1734 (4%)	362 (1%)		**-**

The sequence coverage rate was calculated by considering the number of sequences common to 2, 3 or 4 methods compared to one method chosen as a reference (left row in Table 2). 1938 sequences were selected by all 4 methods, whereas 6721 (1938 sequences added with the 4783 sequences by mm, sdm and sm) sequences are common to mm, sm and sdm. The difference between these 2 values can be explained by the numerous sequences selected only by rm. The mean coverage rates observed for mm and sdm are quite similar at around 75% (10076 sequences specifically selected by these two methods added with the 6721 sequences previously quoted and the 3230 sequences selected by mm, sdm and rm). This similarity might be expected, since both methods are fully automatic and entirely based on the E-values.

We also noticed that the sequences common to mm, sdm and sm sampled sets are usually located at positions in the E-value distribution where large differences occur (see Additional file 1: Sequences selected in the case of the 1QJ4 protein). Thus, we conclude that mm, sm and sdm select mainly variable sequences which may supplement the structural and/or functional information of the sampled set of sequences.

### Impact of the sampling on the potential information in the sequence set

To estimate the impact of the methods on the potential information in the sampled sets, we used multiple alignment of complete sequences (MACS) as the main tool. We analysed the diversity of the sequences included in the MACS, the global quality of the MACS and the extent to which active site residues were observed in conserved columns in the different MACS.

#### Sequence diversity

At the structural and functional level, closely related sequences may not add relevant information, whereas diversity is usually more informative[12]. In the context of Blast searches, sequences detected with nearly the same E-values, especially in the case of low E-values, are more likely to be similar, and inversely, a difference in the E-values usually represents a sequence divergence.

As the information content of a set of homologous sequences is generally related to their diversity, we investigated the SDS (Sampled Distant Sequences), whose selection increases the diversity of the sampled MACS compared to the MACS_init (see *Methods*).

The proportion of SDS selected by the different methods is between 22% for sm and 76% for rm (Table 3). The sm is the method that selects the least SDS because the pre-defined maximal number of sequences inhibits the inclusion of SDS compared to a sampling method that is not limited in terms of the number of sequences. Based on the proportion of SDS (respectively 41 and 46%), we assume that the variability of the mm and sdm sampled MACS is considerably larger than that of MACS_init.

**Table 3:** Proportion of SDS selected by sampling methods

	Total Blast set (*)	**mm**	**sdm**	**sm**	**rm**
**Total number of sequences**	121185	18518	16774	4696	36310
**Number of *SDS***	-	7506	7694	1038	27748
***SDS *proportion (%)**	0	41	46	22	76

* with more than 500 sequences detected by BlastP with E-value ≤ 0.001

#### MACS quality

In order to be informative, a MACS needs to be of high quality. The quality of the alignments used in this study were evaluated using the norMD[13] (normalized Mean Distance) objective function, shown in Table 4. NorMD is an objective function that takes into account *ab initio *sequence information, such as the number, length and similarity of the sequences to be aligned. Of the 284 MACS_init alignments, a total of 225 (79%) had high norMD scores. Sampling by mm, sdm and rm resulted in similar proportions of high quality MACS (83%, 77% and 75% respectively) whereas sm increases this proportion to 95%. As the 284 protein dataset is composed of PDB sequences, it is likely to be enriched in single domain sequences. To verify that the MACS quality was not affected by the presence of multidomain proteins, we compared the norMD scores obtained with and without the multidomain proteins defined according to PFAM[14] annotations of the PDB entries, before and after sampling (see Additional file 2: Proportion of good quality MACS and mean norMD for the 284 protein dataset and restricted to single domain proteins). The norMD scores were similar in all experiments. This observation confirms that the computation of MACS using the combination of local and global algorithms, as implemented in the PipeAlign[15] cascade (see *Methods*), results in high-quality alignments, even in the case of multidomain proteins [16,17]. Furthermore, no major differences were observed between the ROC curves (see 'MACS Information content' below) obtained for the initial 284 protein dataset compared to this reduced dataset of 212 single domain proteins (data not shown).

**Table 4:** Proportion of good quality MACS and mean norMD

	**MACS**	**init**	**mm**	**sdm**	**sm**	**rm**
	
	**Good quality MACS (%)**	79	83	77	95	75
**subset-100 (91 seq.)**	norMD ≥ 0.3 (%)	95	100	98	99	99
	mean norMD*	0.78	0.93	0.95	0.86	0.96
	Standard deviation*	0.42	0.34	0.51	0.41	0.38
**subset100–500 (114 seq.)**	norMD ≥ 0.3 (%)	68	92	83	**96**	82
	mean norMD*	0.51	0.56	0.55	**0.63**	0.60
	Standard deviation*	0.20	0.20	0.17	**0.20**	0.30
**subset+500 (79 seq.)**	norMD ≥ 0.3 (%)	78	49	43	**89**	37
	mean norMD*	0.49	0.46	0.45	**0.53**	0.47
	Standard deviation*	0.16	0.11	0.10	**0.10**	0.17

* considering only good quality MACS

In order to investigate the relationship between MACS quality and the number of sequences detected by BlastP, we also studied the quality of the MACS obtained in the three subsets of comparable size defined in the section *Sequence reduction rate*. For subset-100, 95% of the MACS_init can be considered to be of good quality, with a high mean norMD value (0.78) as shown in Table 4. Sampling the sequences using any of the 4 methods increases both the proportion of good quality MACS (from 98 to 100% compared to 95%) and the mean norMD (from 0.86 to 0.96 compared to 0.78). Similar results were observed for subset100–500, where the sampling methods again increased the proportion of good quality MACS (82 to 96% compared to 68% for MACS_init) and the mean norMD (0.55 to 0.63 compared to 0.51). For subset100–500, sm which is the method that reduces the most the set of aligned sequences, results in a higher proportion of good quality MACS and a higher mean norMD. Furthermore, for subset+500, sm is the only sampling method able to improve the MACS global quality compared to MACS_init, both in terms of proportion (89% compared to 78%) and mean norMD value (0.53 compared to 0.49). It is important to note that the high proportion of SDS added in the context of subset+500 by the mm, sdm and rm methods, corrupts the resulting alignments: the proportion of good quality MACS falls respectively to 49, 43 and 37%. Increasing the sequence diversity in a MACS should normally improve the information content[12], but including too many distant sequences can also be harmful in terms of quality, so that the MACS becomes less informative. This seeming contradiction clearly reflects the current limitations of the algorithms used to construct multiple alignments.

From a quality point of view, we conclude that sm is the most appropriate sampling method since a higher proportion of good quality MACS is obtained after sampling, as well as an increased mean norMD value. Moreover, by significantly reducing the number of sequences to be aligned, the sm method also reduces the computation time required to construct the MACS.

#### MACS information content

The information content of a MACS is difficult to measure objectively. We therefore decided to investigate the residues annotated in the PDB database as being involved in functional active sites. These residues are usually well conserved in a protein family[18,19] and well characterized both biochemically and structurally. As an estimate of the information content of a MACS, we calculated the number of known active sites that were detected in conserved columns of the alignment. Given a conservation threshold cut-off *x*, a column is considered to be "conserved" if *x*% of the residues, including gaps, are identical in the column. The sensitivity and specificity of the active site detection can then be computed (see *Methods*).

In this study, we only considered those tests for which the init, mm, sdm and sm all resulted in good quality MACS, which represents 192 of the 284 protein dataset. The rm method has been excluded from this study based on the results of the MACS quality analysis (see above). In subset+500, low quality MACS were obtained after rm sampling. Furthermore, preliminary analyses of active site detection using rm indicated that the mean sensitivity is much lower compared to all the other methods (see Additional file 3: G-mean results associated with detailed sensitivity and specificity when considering all proteins in subset+500 using the 80% threshold), indicating that the informational content was not conserved.

**Figure 5:**
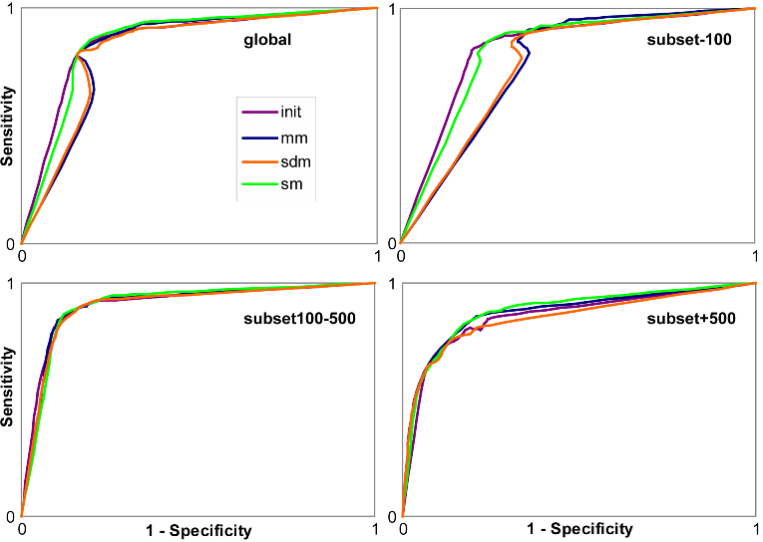
**ROC curves**. Based on the 192 common good quality MACS, ROC curves were constructed for the global protein set, subset-100, subset100–500 and subset+500 based on the residue conservation analysis. ROC curves are coloured according to the sampling method: in violet no sampling method, in blue mm, in orange sdm and in green sm.

In order to compare the informational content of the studied MACS, we plotted the ROC curves for each sampling method using 9 conservation thresholds, ranging from 60% to 100% (Figure 5). Considering the global set of MACS_init alignments, the area under the curve (AUC) is 0.84, indicating that the column conservation measure is a suitable parameter for the discrimination of active site residues. The visual comparison of the AUC shows that the quality of the information is well conserved after sampling, particularly with mm and sm, when considering subset100–500 and subset+500.

**Figure 6:**
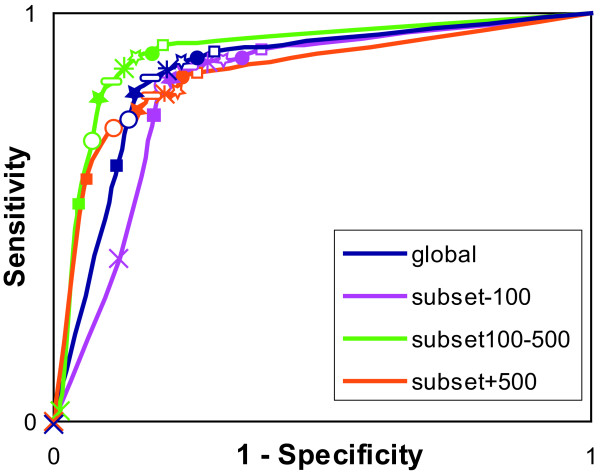
**Determination of the most suitable conservation threshold**. ROC curves based on MACS_init residue conservation analysis for the 192 common good quality MACS: in blue for the global set, in pink for subset-100, in green for subset100–500 and in orange for subset+500. The tested thresholds are represented by: × 100%; ■ 95%; ○ 90%;  85%;  80%;  75%;  70%; ● 65%; □ 60%.

To confirm these observations, we performed a quantitative analysis. We determined the most suitable column conservation threshold for the discrimination of active site residues with MACS_init alignments (Figure 6), corresponding to the inflexion point of the ROC curve near the top and left corner. The threshold for MACS_init for all the 192 good quality MACS is 80%. To assess the information content of the sampled MACS, we constructed the confusion matrix[20] associated with each sampling method, based on this 80% conservation threshold. The geometric means of accuracy (G-mean)[21] values were then computed (Table 5) and the difference in G-means before and after the application of each sampling method was used as a comparison metric. A smaller G-mean would be characteristic of a loss of information content. The G-mean value associated with MACS_init is 0.83: sm is the only sampling method which has the same G-mean value, while a small G-mean decrease is observed for mm and sdm (respectively 0.82 and 0.81).

**Table 5:** G-mean results associated with detailed sensitivity and specificity

		**Se**	**Sp**	**G-mean**
**global **	init	0.83	0.82	0.83
**(threshold = 80%)**	mm	0.86	0.78	0.82
**192 proteins**	sdm	0.83	0.79	0.81
	sm	0.86	0.81	0.83

**subset-100 **	init	0.82	0.80	0.81
**(threshold = 90%) **	mm	0.86	0.67	0.76
**86 proteins**	sdm	0.84	0.69	0.76
	sm	0.81	0.78	0.79

**subset100–500 **	init	0.86	0.87	0.87
**(threshold = 75%) **	mm	0.88	0.85	0.87
**76 proteins**	sdm	0.86	0.86	0.86
	sm	0.89	0.84	0.87

**subset+500 **	init	0.80	0.82	0.81
**(threshold = 80%)**	mm	0.72	0.89	0.80
**30 proteins**	sdm	0.68	0.90	0.78
	sm	0.79	0.85	0.82

We then considered the three subsets defined in the section *Sequence reduction rate *separately, and the corresponding MACS_init ROC curves are shown in Figure 6.

For subset-100, the most suitable column conservation threshold for active site discrimination is 90%. G-mean values decrease with the application of any of the sampling methods: the MACS_init G-mean value (0.81) is slightly reduced after sm (0.79) and a larger reduction is observed after mm and sdm (both 0.76). This decrease is caused by a loss of specificity of the active site detection, directly linked to the reduction of sequence diversity after sampling. Indeed, when only a small number of homologues are detected by BlastP, the variability between the sequences is usually relatively low. Consequently the associated MACS contains a higher proportion of conserved columns, and more false positive predictions are obtained. However, this is not a serious problem as the unsampled MACS_init alignments for this subset are generally of high quality and the small number of sequences (<100) in the BlastP results means that reduction of the sequence set is not necessary for computational purposes.

For subset100–500, the most suitable column conservation threshold is 75%. The highest sensitivity for active site discrimination was obtained using sm sampling (0.89). However, the sensitivity and specificity scores are quite similar for all the sampling methods (G-mean values are all between 0.86 and 0.87) and the differences observed between the methods cannot be considered to be significant. Nevertheless, we observed previously that in this subset, the sm sampling is more accurate in terms of reduction rate and MACS quality, and consequently sm seems to be the most suitable sampling method.

Finally, for subset+500, a 80% conservation threshold was determined. The sensitivity of active site determination after mm and sdm sampling decreases drastically (0.72 and 0.68 respectively compared to 0.80 with no sampling), whereas the sensitivity and specificity of the sm sampled set are both close to the values obtained for MACS_init (Se = 0.79/Sp = 0.85 and Se = 0.80/Sp = 0.82 respectively). This leads to similar G-mean values for MACS_init and mm (0.81 and 0.80 respectively), a small decrease is observed for sdm (0.78), whereas sm shows a better G-mean value (0.82), indicating a better accuracy for active site detection. These observations correlate with the MACS quality results in which the proportion of good quality MACS is higher after application of sm sampling compared to the other methods. The study of sequence coverage showed that the mm and sdm sampled sets both contain a higher proportion of SDS compared to sm (Table 3). Moreover, the sm sampling resulted in a higher reduction rate than the mm and sdm methods under these conditions (Table 1). Without sequence sampling, the average time to construct a multiple alignment for the set of 79 alignments with more than 500 proteins was 995 seconds (maximum time: 4740 seconds). After sampling with the sm method, the average time for the same set of alignments was 17 seconds (maximum time: 125 seconds). Thus, all these observations converge towards the conclusion that sm is the most suitable sampling method for the effective reduction of the number of sequences detected by BlastP, while maintaining the powerful information content of the subsequent MACS.

## Conclusion

The rapid accumulation of numerous homologues in the sequence databases is a problem for which no unique solution exists. This study demonstrates that it is possible to sample the homologous sequences detected by BlastP while at the same time retaining the relevant information concerning the active site residues inside the sampled set of sequences.

We showed that on average 30% of the detected sequences are sufficient to efficiently maintain the relevant functional information, however the sequence selection cannot be performed randomly.

An exhaustive analysis allowed us to define the most suitable sampling strategy depending on the number of sequences detected by BlastP searches and in conjunction with the use of a non redundant database:

1. The reduction of the sequence set is not necessary with proteins having few homologues in sequence databases (less than 100). In this case, the variability between the sequences is usually relatively low and sampling the sequences results in a loss of information.

2. The strips sampling (sm) is the most suitable sampling method for the effective reduction of the sequence set when more than 100 sequences are detected by BlastP searches. This method maintains the potential structural and functional information in the sampled set and by defining the maximal number of sequences (set here to 100) the computation time remains reasonable.

In conclusion, regardless of the size of the initial BlastP results, our sampling strategy produces a set of sequences that is computationally and humanly manageable.

In the future, we will study the conservation of other kinds of information that can be extracted from a set of homologous sequences, such as secondary structure information or motif conservation.

## Methods

### 284 protein dataset

We defined a set of 284 distinct proteins, which we refer to as the "284 protein dataset" using a similar methodology to that developed by Aloy and co-workers[22] for the creation of a protein test set for the prediction of functional sites. To build our 284 protein dataset, we selected protein sequences sharing less than 70% identity with one another and having an annotated active site, from the March 2005 release of the PDB[11]. When several polypeptide chains existed for a single PDB entry, the chain containing the most annotated catalytic residues was selected first, and then the longest one. The information concerning the active site residues was extracted from the SITE records description when "*active*" or "*catalytic*" words were found in the associated definition. The 284 proteins consisted of a total of 96403 residues, of which 1045 represented active site residues (from 1 to 20 residues per protein).

The 284 protein dataset covers a large part of the protein fold space according to the CATH[23] classification. Only 9 proteins have not been classified and 126 proteins have been defined as multi-domain proteins. 440 domains are represented, of which 68 belong to class 1 (mainly alpha),119 to class 2 (mainly beta), 251 to class 3 (mixed alpha-beta) and 2 to class 4 (few secondary structures).

These 284 proteins correspond to 257 enzymes and 27 non-enzymes. According to the official Enzyme Nomenclature[24], they can be classified as follows: 49 oxidoreductases (EC 1), 26 transferases (EC 2), 148 hydrolases (EC 3), 23 lyases (EC 4), 9 isomerases (EC 5) and 2 ligases (EC 6). The non-enzyme proteins are mostly toxins, binding proteins and inhibitors.

The full list of PDB names is available (see Additional file 4: List of the PDB identifier constituting the 284 protein dataset).

### BlastP searches

The BlastP searches were performed on the UniRef90 database[7] (2005/05/23 version), a non redundant database based on UniProt[25], for which sequences sharing more than 90% identity are clustered in one single entry corresponding to a representative sequence for this cluster. We chose a non redundant database in order to avoid the over-representation of identical or nearly identical sequences resulting from closely related genome sequencing projects, etc. Such very closely related sequences were ignored as they do not add any significant information in terms of catalytic functional residues. The standard version 2.2.10 of BlastP[26] has been used, and parameters e, v, and b were set to 0.001, 5000 and 5000 respectively, allowing the retrieval of up to 5000 sequences and alignments.

### Characterisation of the BlastP E-value distribution

All the 153128 sequences detected with E-value ≤ 0.001 by BlastP for the 284 protein dataset were pooled and sorted according to their respective E-values. This list was then divided into 10 equally populated intervals and the E-values corresponding to the boundaries of each interval were defined from this cutting (interval 1: 1.10^-200 ^to 4.10^-67^; interval 2: 4.10^-67 ^to 1.10^-39^; interval 3: 1.10^-39 ^to 7.10^-30^; interval 4: 7.10^-30 ^to 1.10^-23^; interval 5: 1.10^-23 ^to 1.10^-18^; interval 6: 1.10^-18 ^to 2.10^-14^; interval 7: 2.10^-14 ^to 6.10^-11^; interval 8: 6.10^-11 ^to 8.10^-8^; interval 9: 8.10^-8 ^to 2.10^-5^; interval 10: 2.10^-5 ^to 0.001). For each individual BlastP result, the percentage of sequences in each interval was calculated and we thus obtained for each BlastP, a list of 10 values ranging from 0 to 100% characterising the E-value distribution for this BlastP search.

The 284 lists were then clustered using two classification programs: a Dirichlet mixture algorithm[27] and the Secator program[28]. The same global tendencies were observed with both methods with different degrees of resolution (data not shown). We choose to work with Secator's classification which avoided the creation of poorly populated groups. The chosen classification resulted in 10 groups that we named E-clusters (Figure 3). E-cluster 1 corresponds to a highly populated interval 1, i.e. a majority of highly similar sequences detected by BlastP. E-cluster 10 corresponds to a highly populated interval 10, i.e. the BlastP result contains a majority of weakly related sequences.

### MACS construction

A tuned version of the PipeAlign program suite[15] has been used for the computation of high quality MACS. PipeAlign offers an integrated approach for protein family analysis allowing the following steps to be automatically achieved:

- Ballast[29] processes the Blast results and determines anchors, called LMS (Local Maximum Segments) based on the high scoring-segment pairs detected by Blast.

- DbClustal[30] uses the LMS as anchors to create a MACS. Sequence fragments are eliminated, and the number of sequences to be aligned is limited to 500 (corresponding to the 500 lowest E-values before or after sampling).

- Rascal[31] scans the complete alignment and corrects locally misaligned regions.

- norMD[13] objectively estimates the MACS quality. It combines the advantages of a column-scoring technique with the sensitivity of methods incorporating residue similarity scores. It also incorporates gap information and *ab initio *sequence information, such as the number, length and similarity of the aligned sequences. A norMD score ≥ 0.3 is assumed to indicate a good quality alignment.

### Sampling methods

The 4 sampling methods studied here select sequences having an E-value ≤ 0.001, which is the threshold commonly associated to a significant homology. The sampling methods can be classified in two categories:

- automatic methods:

- the mean method (mm). A threshold is defined as the mean difference between successive E-value logarithms[32]. Let n be the number of sequences detected with an E-value ≤ 0.001, E_n _is the E-value associated with the n^th ^sequence and E_1 _the lowest printed E-value.

Threshold=log⁡(En)−log⁡(E1)n−1
 MathType@MTEF@5@5@+=feaafiart1ev1aaatCvAUfKttLearuWrP9MDH5MBPbIqV92AaeXatLxBI9gBaebbnrfifHhDYfgasaacH8akY=wiFfYdH8Gipec8Eeeu0xXdbba9frFj0=OqFfea0dXdd9vqai=hGuQ8kuc9pgc9s8qqaq=dirpe0xb9q8qiLsFr0=vr0=vr0dc8meaabaqaciaacaGaaeqabaqabeGadaaakeaacqqGubavcqqGObaAcqqGYbGCcqqGLbqzcqqGZbWCcqqGObaAcqqGVbWBcqqGSbaBcqqGKbazcqGH9aqpdaWcaaqaaiGbcYgaSjabc+gaVjabcEgaNnaabmaabaGaemyrau0aaSbaaSqaaiabd6gaUbqabaaakiaawIcacaGLPaaacqGHsislcyGGSbaBcqGGVbWBcqGGNbWzdaqadaqaaiabdweafnaaBaaaleaacqaIXaqmaeqaaaGccaGLOaGaayzkaaaabaGaemOBa4MaeyOeI0IaeGymaedaaaaa@4E3F@

**Figure 7:**
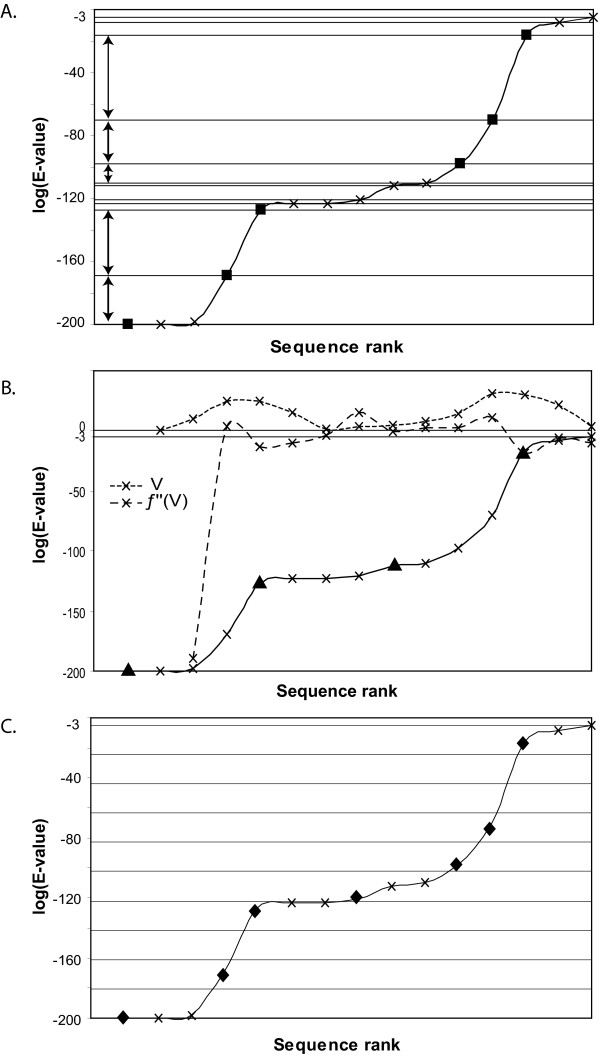
**Sequence selection according to the sampling method algorithms**. Sequences detected by BlastP searches are represented according to the logarithm of their E-value on the graphs (×) and sequences selected by each method are represented by ■ for mm, ▲ for sdm and ◆ for sm. (a) mm selection: differences between the logarithms of 2 successive E-values greater than the computed threshold are represented by a bidirectional arrow; (b) sdm selection: V is the computed E-value variation function. Sequences corresponding to V inflexion points are selected; (c) sm selection: the logarithmic curve of the BlastP E-values is cut in x strips (x = 10 on the graph) of equal width. Inside each non empty strip, the sequence associated to the smallest E-value is selected. mm, sdm and sm systematically select the first sequence detected by BlastP search.

Then, sequence i will be selected if the difference between log(E_i_) and log(E_i+1_) is greater than or equal to this threshold (Figure 7(a)).

- the second derivative method (sdm). The second derivative of the E-value as a function of rank is computed and the sequences corresponding to its inflexion points are selected. Let V be the variation function of the E-value curve, i.e. V_(i+1) _= E_i+1 _- E_i_. A sequence is selected if *f*" (V_i_) < 0 (Figure 7(b)).

These two methods depend entirely on the BlastP E-values and are representative of the E-value dispersion. No limit is given for the number of sequences to be selected.

- customisable methods:

- the strips method (sm), for which the maximal number of sequences to be selected is fixed. The logarithmic graph of the Blast E-values is divided into a preset number x of strips of equal width (x = 100 in this study). Let E_1 _be the smallest E-value from the Blast search results and E_n _the highest one (E_n _≤ 0.001).

Width = 1x
 MathType@MTEF@5@5@+=feaafiart1ev1aaatCvAUfKttLearuWrP9MDH5MBPbIqV92AaeXatLxBI9gBaebbnrfifHhDYfgasaacH8akY=wiFfYdH8Gipec8Eeeu0xXdbba9frFj0=OqFfea0dXdd9vqai=hGuQ8kuc9pgc9s8qqaq=dirpe0xb9q8qiLsFr0=vr0=vr0dc8meaabaqaciaacaGaaeqabaqabeGadaaakeaadaWcaaqaaiabigdaXaqaaiabdIha4baaaaa@2F25@(log(*E*_1_) - log(*E*_*n*_))

Inside each non-empty strip, the sequence associated with the smallest E-value is selected (Figure 7(c)).

- the random method (rm), for which the associated reduction rate was defined as 70%, after initial analysis of the mean reduction rates of the 3 other sampling methods. Sequences are randomly selected on this basis. This method is a control in our study, used to estimate the relevance of a selection according to the sequence dispersion.

### Evaluation of the sequence sets selected by the 4 sampling methods

Two different tests were designed to evaluate and compare the set of sequences selected by each of the four different sampling methods.

#### Sequence reduction rate

The reduction rate estimates the relationship between the total number of sequences detected by a BlastP search with an E-value ≤ 0.001 and the reduced number of sequences after sampling. For each query protein X of the 284 protein dataset:

reductionRate(X)=100−NumberOfSequencesAfterSamplingNumberOfSequencesAfterBlastP(E−value≤0.001)×100
 MathType@MTEF@5@5@+=feaafiart1ev1aaatCvAUfKttLearuWrP9MDH5MBPbIqV92AaeXatLxBI9gBaebbnrfifHhDYfgasaacH8akY=wiFfYdH8Gipec8Eeeu0xXdbba9frFj0=OqFfea0dXdd9vqai=hGuQ8kuc9pgc9s8qqaq=dirpe0xb9q8qiLsFr0=vr0=vr0dc8meaabaqaciaacaGaaeqabaqabeGadaaakeaacqqGYbGCcqqGLbqzcqqGKbazcqqG1bqDcqqGJbWycqqG0baDcqqGPbqAcqqGVbWBcqqGUbGBcqqGsbGucqqGHbqycqqG0baDcqqGLbqzcqGGOaakcqqGybawcqGGPaqkcqGH9aqpcqaIXaqmcqaIWaamcqaIWaamcqGHsisldaWcaaqaaiabd6eaojabdwha1jabd2gaTjabdkgaIjabdwgaLjabdkhaYjabd+eapjabdAgaMjabdofatjabdwgaLjabdghaXjabdwha1jabdwgaLjabd6gaUjabdogaJjabdwgaLjabdohaZjabdgeabjabdAgaMjabdsha0jabdwgaLjabdkhaYjabdofatjabdggaHjabd2gaTjabdchaWjabdYgaSjabdMgaPjabd6gaUjabdEgaNbqaaiabd6eaojabdwha1jabd2gaTjabdkgaIjabdwgaLjabdkhaYjabd+eapjabdAgaMjabdofatjabdwgaLjabdghaXjabdwha1jabdwgaLjabd6gaUjabdogaJjabdwgaLjabdohaZjabdgeabjabdAgaMjabdsha0jabdwgaLjabdkhaYjabdkeacjabdYgaSjabdggaHjabdohaZjabdsha0jabdcfaqjabcIcaOiabdweafjabgkHiTiabdAha2jabdggaHjabdYgaSjabdwha1jabdwgaLjabgsMiJkabicdaWiabc6caUiabicdaWiabicdaWiabigdaXiabcMcaPaaacqGHxdaTcqaIXaqmcqaIWaamcqaIWaamaaa@A8BC@

For a given sampling method, the mean reduction rate is the average of the 284 individual reduction rates:

Mean Reduction rate=1284∑n=1284reductionRate(n)
 MathType@MTEF@5@5@+=feaafiart1ev1aaatCvAUfKttLearuWrP9MDH5MBPbIqV92AaeXatLxBI9gBaebbnrfifHhDYfgasaacH8akY=wiFfYdH8Gipec8Eeeu0xXdbba9frFj0=OqFfea0dXdd9vqai=hGuQ8kuc9pgc9s8qqaq=dirpe0xb9q8qiLsFr0=vr0=vr0dc8meaabaqaciaacaGaaeqabaqabeGadaaakeaacqqGnbqtcqqGLbqzcqqGHbqycqqGUbGBcqqGGaaicqqGsbGucqqGLbqzcqqGKbazcqqG1bqDcqqGJbWycqqG0baDcqqGPbqAcqqGVbWBcqqGUbGBcqqGGaaicqqGYbGCcqqGHbqycqqG0baDcqqGLbqzcqGH9aqpdaWcaaqaaiabigdaXaqaaiabikdaYiabiIda4iabisda0aaadaaeWbqaaiabdkhaYjabdwgaLjabdsgaKjabdwha1jabdogaJjabdsha0jabdMgaPjabd+gaVjabd6gaUjabdkfasjabdggaHjabdsha0jabdwgaLjabcIcaOiabd6gaUjabcMcaPaWcbaGaemOBa4Maeyypa0JaeGymaedabaGaeGOmaiJaeGioaGJaeGinaqdaniabggHiLdaaaa@6712@

#### Sequence coverage

The sampling method coverage at the sequence level (coverage rate) corresponds to the number of sequences selected in common by the sampling methods. The sequence coverage was calculated by considering the number of sequences jointly selected by 2, 3 or 4 methods compared to one method chosen as the reference.

### Evaluation of the MACS information content

Three tests were designed to evaluate the potential information content associated with a MACS.

#### Sampled distant sequences

**Figure 8:**
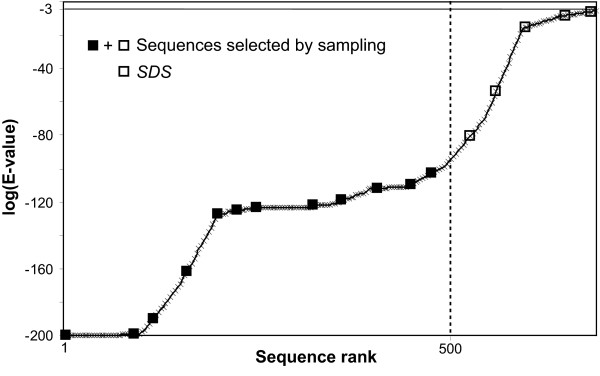
**Sampled distant sequences**. The graph represents sequences collected by BlastP searches according to the logarithm of their E-values. Sequences selected by sampling are marked with a square. When more then 500 sequences are highlighted, the first 500 sequences are aligned in MACS_init. Sampled Distant Sequences (SDS) indicate sequences with an E-value > 0.001 and ranking after the 500 first sequences in BlastP results, selected by any sampling method.

When more than 500 sequences with an E-value ≤ 0.001 are detected by Blast searches, the sampling methods may select sequences that are not present in the original MACS_init. These sequences located after the top 500 (Figure 8) are defined as Sampled Distant Sequences (SDS). By maximising the sequence diversity inside the associated MACS, SDS may add relevant information to the sampled set of sequences.

#### MACS quality

The quality of the test alignments used in this study was evaluated using the norMD[13] (normalized Mean Distance) objective function. As stated in previous studies, a norMD score greater than 0.3 indicates a good quality MACS.

#### Identification of active site residues

In order to estimate the impact of each sampling method on the structural information content of a MACS, we determined the number of active site residues that were found in conserved columns in the MACS. We tested 9 different conservation thresholds: 60%, 65%, 70%, 75%, 80%, 85%, 90%, 95% and 100%. Let x be the considered threshold: a column is considered as a "conserved column" when at least x% of the residues (including gaps) are identical at this position.

##### i) Sensitivity-Specificity

The sensitivity (Se) and the specificity (Sp) of each sampling method are defined as:

Se=TPTP+FNSp=TNTN+FP
 MathType@MTEF@5@5@+=feaafiart1ev1aaatCvAUfKttLearuWrP9MDH5MBPbIqV92AaeXatLxBI9gBaebbnrfifHhDYfgasaacH8akY=wiFfYdH8Gipec8Eeeu0xXdbba9frFj0=OqFfea0dXdd9vqai=hGuQ8kuc9pgc9s8qqaq=dirpe0xb9q8qiLsFr0=vr0=vr0dc8meaabaqaciaacaGaaeqabaqabeGadaaakeaafaqabeqacaaabaGaee4uamLaeeyzauMaeyypa0ZaaSaaaeaacqWGubavcqWGqbauaeaacqWGubavcqWGqbaucqGHRaWkcqWGgbGrcqWGobGtaaaabaGaee4uamLaeeiCaaNaeyypa0ZaaSaaaeaacqWGubavcqWGobGtaeaacqWGubavcqWGobGtcqGHRaWkcqWGgbGrcqWGqbauaaaaaaaa@4393@

Where:

- TP (True Positive) = number of active site residues in conserved columns,

- FP (False Positive) = number of non-active site residues in conserved columns,

- TN (True Negative) = number of non-active site residues in non-conserved columns,

- FN (False Negative) = number of active site residues in non-conserved columns.

##### ii) ROC curve

A Receiver Operating Characteristic curve[33] (ROC curve) is a graph of the true positive rate (sensitivity) versus the false positive rate (1 – specificity), while varying the conservation threshold. It measures the potential of a classifier to discriminate between the two classes, and allows the determination of the most suitable threshold for discrimination: the inflexion of the ROC curve near the top and left axis corresponds to the best classifier performance. Thus, the area under the ROC curve (AUC) provides a single metric that can be used to judge the overall discriminative ability of a classification method. An AUC of 0.5 indicates a random prediction; between 0.7 and 0.8 indicates acceptable discrimination; between 0.8 and 0.9 indicates excellent discrimination, and above 0.9 indicates outstanding discrimination[34].

The most suitable conservation threshold for the discrimination of active site residues was determined by computing ROC curves while varying the classification threshold from 60 to 100% in the context of the MACS_init.

##### iii) G-mean accuracy

To assess the relevance of the sampling methods in terms of MACS information content, we compared the sensitivity and specificity results obtained with and without sampling. We studied the so-called confusion matrix[20], which includes predicted and true active site classifications, and from which several metrics can be obtained. We have "imbalanced classes" in this study: i.e. columns containing active site residues represent only a small minority of the total number of MACS columns, which means that we cannot use metrics such as accuracy or precision which are not suitable for this kind of data. We therefore used the geometric mean of accuracies[21] as a comparison metric defined as:

G-mean=Se×Sp
 MathType@MTEF@5@5@+=feaafiart1ev1aaatCvAUfKttLearuWrP9MDH5MBPbIqV92AaeXatLxBI9gBaebbnrfifHhDYfgasaacH8akY=wiFfYdH8Gipec8Eeeu0xXdbba9frFj0=OqFfea0dXdd9vqai=hGuQ8kuc9pgc9s8qqaq=dirpe0xb9q8qiLsFr0=vr0=vr0dc8meaabaqaciaacaGaaeqabaqabeGadaaakeaacqqGhbWrcqqGTaqlcqqGTbqBcqqGLbqzcqqGHbqycqqGUbGBcqGH9aqpdaGcaaqaaiabdofatjabdwgaLjabgEna0kabdofatjabdchaWbWcbeaaaaa@3C52@

## List of abbreviations

MACS, multiple alignment of complete sequences; PDB, protein data bank; SDS, sampled distant sequences; ROC, receiver operating characteristic; AUC, area under the curve; TP, true positives; FP, false positives; TN, true negatives; FN, false negatives.

## Authors' contributions

AF performed the computational experiments, analyzed them and wrote the manuscript. RR and LM developed respectively the strips and the second derivative methods, and contributed to the experimental work. LM also edited the manuscript and supervised the project. NG, EB and GD provided the mean method and suggested ideas. OP initiated the project, suggested ideas and edited the manuscript. All authors read and approved the final manuscript.

## Supplementary Material

Additional file 1**Sequences selected in the case of the 1QJ4 protein**. Sequences detected by BlastP searches () are represented by their E-values on the graph. Sequences selected by each sampling method are schematized by their projection on horizontal axes above the graph.Click here for file

Additional file 2 **Proportion of good quality MACS and mean norMD for the 284 protein dataset and restricted to the single domain proteins**. These tables summarize the proportion of good quality MACS and mean norMD obtained: – for the 284 protein dataset. – for the 212 single PFAM domain proteins. Click here for file

Additional file 3 G-mean results associated with detailed sensitivity and specificity when considering all proteins in subset+500 Click here for file

Additional file 4 **List of the PDB identifier constituting the 284 protein dataset**. In this list, the chain identifier corresponds to the last character, with "_" denoting no chain. Click here for file
