# Supplementary figures and images for: Blast sampling for structural and functional analyses
Source: BMC Bioinformatics. 2007 Feb 23;8:62. doi: 10.1186/1471-2105-8-62 (PMC1819393; doi:10.1186/1471-2105-8-62)

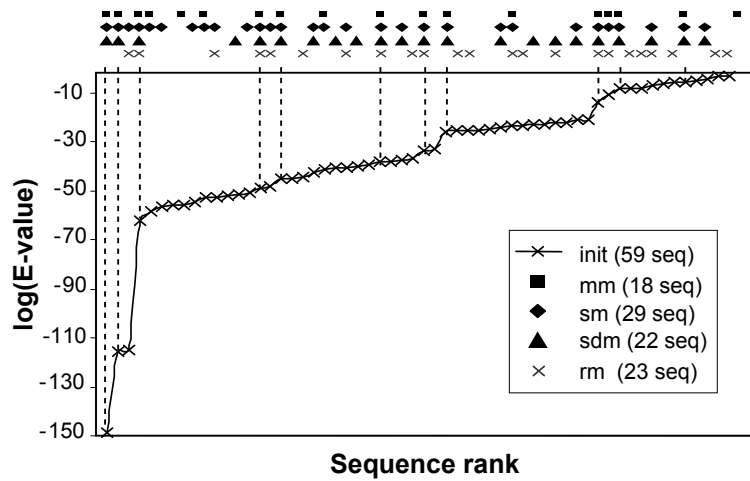

Supplement: Additional file 1 — Sequences selected in the case of the 1QJ4 protein. Sequences detected by BlastP searches () are represented by their E-values on the graph. Sequences selected by each sampling method are schematized by their projection on horizontal axes above the graph. [file 1471-2105-8-62-S1.pdf]
