# Supplementary material for: Blast sampling for structural and functional analyses
Source: BMC Bioinformatics. 2007 Feb 23;8:62. doi: 10.1186/1471-2105-8-62 (PMC1819393; doi:10.1186/1471-2105-8-62)
Supplement: Additional file 2 — Proportion of good quality MACS and mean norMD for the 284 protein dataset and restricted to the single domain proteins. These tables summarize the proportion of good quality MACS and mean norMD obtained: – for the 284 protein dataset. – for the 212 single PFAM domain proteins. [file 1471-2105-8-62-S2.pdf]

Initial dataset, 284 seq	**init**	**mm**	**sm**	**sdm**	**rm**
**norMD>0,3 (%)**	79	83	95	77	75
**Mean norMD > 0,3**	0,61	0,69	0,69	0,71	0,73
**EC norMD > 0,3**	0,33	0,32	0,3	0,41	0,38

Single PFAM domain, 212 seq	**init**	**mm**	**sm**	**sdm**	**rm**
**norMD>0,3 (%)**	80	85	95	79	77
**Mean norMD > 0,3**	0,63	0,71	0,7	0,72	0,75
**EC norMD > 0,3**	0,35	0,34	0,33	0,44	0,39
