# Supplementary material for: Blast sampling for structural and functional analyses
Source: BMC Bioinformatics. 2007 Feb 23;8:62. doi: 10.1186/1471-2105-8-62 (PMC1819393; doi:10.1186/1471-2105-8-62)
Supplement: Additional file 3 — G-mean results associated with detailed sensitivity and specificity when considering all proteins in subset+500 [file 1471-2105-8-62-S3.pdf]

		Se	Sp	G-mean
**subset+500 (threshold = 80%) 79 proteins**	init	0.79	0.85	0.82
	mm	0.74	0.91	0.82
	sdm	0.68	0.91	0.79
	sm	0.84	0.86	0.85
	rm	0.57	0.92	0.72
